# Implementation of a central-line bundle: a qualitative study of three clinical units

**DOI:** 10.1186/s43058-021-00204-y

**Published:** 2021-09-16

**Authors:** Joanne Goldman, Leahora Rotteau, Kaveh G. Shojania, G. Ross Baker, Paula Rowland, Marlys K. Christianson, Timothy J. Vogus, Connie Cameron, Maitreya Coffey

**Affiliations:** 1 Centre for Quality Improvement and Patient Safety, Temerty Faculty of Medicine, University of Toronto, 630-525 University Ave., Toronto, M5G2L3 Canada; 2 Department of Medicine, Temerty Faculty of Medicine, University of Toronto, Toronto, Canada; 3 Wilson Centre for Research in Education, University of Toronto, 200 Elizabeth St., 1ES-565, Toronto, M5G 2C4 Canada; 4 Division of General Internal Medicine, Sunnybrook Health Sciences Centre, Toronto, Canada; 5 Institute of Health Policy, Management and Evaluation, University of Toronto, Health Sciences Building, 155 College St., Suite 425, Toronto, M5T 3M6 Canada; 6 Department of Occupational Science and Occupational Therapy, Temerty Faculty of Medicine, University of Toronto, Toronto, Canada; 7 Rotman School of Management, University of Toronto, 125 St. George St., Toronto, M5S 2E8 Canada; 8 Owen Graduate School of Management, Vanderbilt University, 401 21st Avenue South, Nashville, TN 37203 USA; 9 The Hospital for Sick Children, 555 University Ave., Toronto, M5G 1X8 Canada; 10 Department of Paediatrics, University of Toronto, Toronto, Canada; 11 Children’s Hospitals Solutions for Patient Safety, Cincinnati, OH USA

**Keywords:** Central-line bundle, Central-line-associated bloodstream infections, Implementation, Qualitative research, Patient safety

## Abstract

**Background:**

Evidence for the central line-associated bloodstream infection (CLABSI) bundle effectiveness remains mixed, possibly reflecting implementation challenges and persistent ambiguities in how CLABSIs are counted and bundle adherence measured. In the context of a tertiary pediatric hospital that had reduced CLABSI by 30% as part of an international safety program, we aimed to examine unit-based socio-cultural factors influencing bundle practices and measurement, and how they come to be recognized and attended to by safety leaders over time in an organization-wide bundle implementation effort.

**Methods:**

We used an interpretivist qualitative research approach, based on 74 interviews, approximately 50 h of observations, and documents. Data collection focused on hospital executives and safety leadership, and three clinical units: a medical specialty unit, an intensive care unit, and a surgical unit. We used thematic analysis and constant comparison methods for data analysis.

**Results:**

Participants had variable beliefs about the central-line bundle as a quality improvement priority based on their professional roles and experiences and unit setting, which influenced their responses. Nursing leaders were particularly concerned about CLABSI being one of an overwhelming number of QI targets for which they were responsible. Bundle implementation strategies were initially reliant on unit-based nurse education. Over time there was recognition of the need for centralized education and reinforcement tactics. However, these interventions achieved limited impact given the influence of competing unit workflow demands and professional roles, interactions, and routines, which were variably targeted in the safety program. The auditing process, initially a responsibility of units, was performed in different ways based on individuals’ approaches to the process. Given concerns about auditing reliability, a centralized approach was implemented, which continued to have its own variability.

**Conclusions:**

Our findings report on a contextualized, dynamic implementation approach that required movement between centralized and unit-based approaches and from a focus on standardization to some recognition of a role for customization. However, some factors related to bundle compliance and measurement remain unaddressed, including harder to change socio-cultural factors likely important to sustainability of the CLABSI reductions and fostering further improvements across a broader safety agenda.

Contributions to the literature

- Many have highlighted the importance of socio-cultural factors in central-line-associated bloodstream infection bundle implementation. Yet limited evidence provides specific guidance on how to consider or act on such factors.
- We provide insight into the ongoing adjustments in implementation approaches that occurred in response to variability in factors including beliefs, workflows, interprofessional interactions, and measurement practices related to the bundle across three units.
- Movements between central versus unit, and standardized versus customized, implementation approaches can explain successful measured outcomes.
- Not all behaviors related to bundle compliance and measurement, though, were addressed, which may have implications for broader safety agendas.

## Background

The concept of the care bundle as a quality improvement (QI) tool emerged in 2001 from a joint initiative between the Institute for Healthcare Improvement (IHI) and the Voluntary Hospital Association. This initiative included a bundle to reduce central-line-associated bloodstream infections (CLABSI) [1]. A care bundle refers to a “small set of evidence-based interventions … that, when implemented together, will result in significantly better outcomes than when implemented individually (p. 2)” [1]. The Michigan Keystone ICU project reported substantial reductions (up to 66%), with some participating intensive care units (ICUs) achieving virtual eradication of CLABSI [2]. However, mixed results in subsequent studies have contributed to divergent perspectives about a goal of “zero CLABSI” and the prioritization of resources for this pursuit [3–12].

Reflecting on the Michigan project, Bosk et al. [13] emphasized that the care bundle constituted just one component of a more comprehensive program to alter the culture of ICUs. They argued that a technical solution (bundle) would not succeed without simultaneous attention to socio-cultural changes required to support adherence to the recommended practices. They also noted that bundle implementation work was “arduous and often laden with emotions” (p. 444). Other research clearly demonstrates the influences on central-line bundle implementation from socio-cultural factors, such as divergent implementation agendas, level of organizational commitment, physician and nurse engagement, and meaningful use of data among others [14–20]. However, how particular factors come to the forefront during bundle implementation in different clinical contexts and how they are, or are not, addressed over time remains unclear.

This lack of clarity about the ways specific socio-cultural factors exert their effects may explain the observed variability in bundle adherence and outcomes achieved across efforts to implement the CLABSI bundle [6, 21]. The expanded scope of such efforts—from ICUs to a range of adult and pediatric hospital units—and the increasing inclusion of CLABSI rates among organization-wide safety targets [10, 20, 22–26], underscores the need for greater understanding of the bundle implementation processes across a range of contexts. In this paper, we report a qualitative study of the CLABSI bundle implementation efforts in three clinical units in a pediatric hospital participating in a hospital-wide patient safety program. We sought to examine how socio-cultural factors influencing bundle implementation and practices in these units came to be recognized and attended to by leaders of this safety program.

## Methods

The findings reported in this paper are part of a larger qualitative study of the implementation of a hospital-wide safety program. The safety program, part of the international Children’s Hospitals’ Solutions for Patient Safety (SPS) Network [27–29], aimed to enhance the hospital’s safety culture and outcomes by adhering to the principles of high reliability organizations. The safety program, launched in 2015, consisted of a range of interventions, including the targeting of seven hospital-acquired conditions (HACs), one being CLABSI. CLABSI was targeted given its prevalence and association with high morbidity and healthcare costs [27, 30]. SPS collects and shares hospital outcome and process data [31, 32]. This paper reports on our data related to CLABSI bundle implementation. We used an interpretivist qualitative research approach to understand participants’ understandings and experiences of the bundle and its practices [33].

### Study setting and context

The study was conducted at a 300-bed tertiary academic pediatric hospital in Canada. SPS provided a CLABSI bundle developed using evidence from the medical literature as well as testing and analysis to identify practices highly likely to result in decreased harm [34]. The bundle included “standard” (required, supported by strongest evidence) and “recommended” (encouraged, supported by weaker evidence) elements (Table 1). For process reliability measurement, each unit was required to complete a minimum of 20 audits per month, using an all-or-none approach (bundle complete or not) to monitor for compliance and inform implementation. For outcome measurement, the hospital utilized the National Healthcare Safety Network (NHSN) definition as used by SPS [35]. This definition includes all CLABSI, including mucosal barrier infection (MBI) CLABSI, which occur commonly in certain patient populations (e.g., immunosuppressed patients receiving cancer treatment) and are not expected to decrease even with optimal implementation of and adherence to the bundle [36]. The hospital reported total CLABSI data to SPS, yet internal targets focused on non-MBI CLABSI. Over time, recognition of improvements in MBI CLABSI rates, both internal to the hospital and in the larger network, led to increasing attention to total (non-MBI and MBI combined) CLABSI rates.

**Table 1:** CLABSI bundle recommendations (Version 3, Date 12/30/2015)

SPS Standard elements	Daily discussion of line necessity, functionality and utilization including bedside and medical care team members Regular assessment of dressing to assure clean/dry/occlusive Standardized access procedure Standardized dressing, cap and tubing change procedures/timing
SPS Recommended elements	An in-depth review of all identified CLABSI with multidisciplinary involvement AND the intent to change the process if needed Daily chlorhexidine gluconate bathing and linen changes

**Fig. 1:**
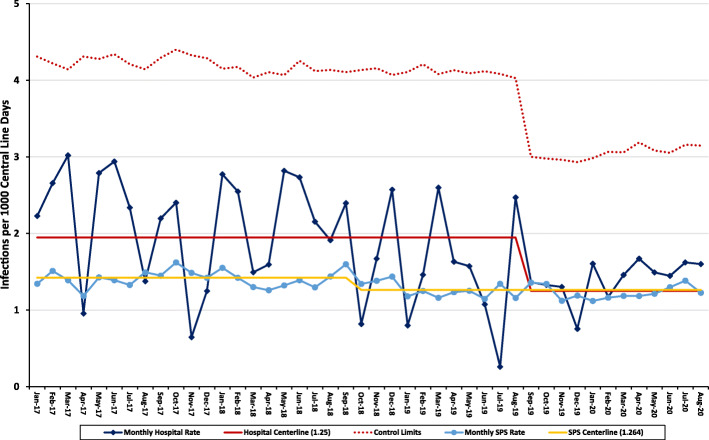
Statistical process control chart: hospital CLABSI rate outcomes over time

The hospital used standardized methods for CLABSI surveillance already in place in select areas but spread hospital-wide over the course of the project. Infection prevention and control practitioners utilized blood culture data as a trigger to identify potential CLABSI, and then performed chart review to assess whether the case met the NHSN surveillance definition. Determination of the denominator (central line days) initially involved a combination of electronic and manual methods, eventually becoming a unified process via the comprehensive electronic health record. Each of the units under study had different baseline CLABSI rates, and each achieved improvement over the 2017–2019 period, contributing to an overall hospital-wide reduction of 30% (Fig. 1).

Table 2 provides an overview of project chronology and results obtained as contextual background for this qualitative study.

**Table 2:** Overview of CLABSI program timeline and outcome results

CLABSI program timeline		
Year	Activity	Results
**2015**	Hospital joined safety network, formed steering committee, attended network education sessions, and conducted extensive corporate communications regarding initiative.	CLABSI outcome data submission to network initiated (ICUs only)
**2016**	CLABSI education and unit-based auditing initiated (three ICUs only), began collection of outcomes data by unit.	Performance relative to external comparators and change in outcomes over time was variable across ICUs
**2017**	CLABSI education and unit-based auditing spread hospital-wide. Outcome data available for all units.	Performance relative to comparators and change over time was variable among units (Pediatric ICU, Neonatal ICU, and Cardiac ICU, general medical, surgical, and specialty units)
**2018**	Hospital-wide 1 year 10% non-MBI CLABSI reduction goal, and three-year 30% HAC reduction goal (including CLABSI) established. New central HAC audit team created to increase audit reliability hospital-wide. Audits performed by both central team and unit-based leaders. CLABSI oversight committee created and leadership rounding initiated.	Hospital level goals not achieved (variable changes in outcomes across units)
**2019**	Expanded hospital CLABSI committees to include additional CLABSI physician group, passive disinfecting caps implemented, mandatory unit-leadership Aseptic Non-Touch Technique (ANTT) sessions completed and ANTT e-learning module delivered to all unit-based nurses.	Hospital-wide 10% non-MBI CLABSI reduction goal exceeded
**2020**	Continued 2019 interventions and spread of in-person, unit-based ANTT education sessions.	Sustained rate of improvement, resulting in a 30% reduction in total CLABSI hospital-wide (using statistical process control, centreline shifted from 1.9 to 1.3 CLABSI per 1000 line days)

### Data collection and analysis

We collected data from April 2017 through February 2019 using interview, observation, and documentary data collection methods. Data collection focused on hospital executives and safety leadership, and three clinical units: a medical specialty unit, an ICU, and a surgical unit. We chose units purposefully to reflect diversity in acuity and specialization, medical vs. surgical care, and earlier vs. later CLABSI bundle implementation.

We conducted one-on-one, and in two cases, small group, semi-structured interviews to gain insight into participants’ perceptions and experiences with the safety program. Interview recruitment strategies were directed at hospital executives and safety leaders, and the varied members of each clinical unit. Members of these groups were sent an e-mail explaining the study and inviting them to follow up with the researchers if they were interested in participating in an interview. For each clinical unit, we aimed for a purposeful maximum variation sampling approach [37], to include individuals with a range of professional and administrative backgrounds and roles. All individuals who volunteered were interviewed. Informed consent was obtained from all those who agreed to be interviewed prior to the start of the interview.

The interview guide included questions about participants’ perspectives and experiences with the safety program, high reliability principles, and HAC prevention efforts. In early interviews, participants talked considerably more about CLABSI than other HACs, so we probed further about CLABSI in subsequent interviews. The interview guide was adapted to each participant’s background based on the individual’s role with respect to CLABSI (e.g., safety leader, nurse, auditor). Interview guides covered perceptions of the bundle and its implementation, perceived challenges and facilitators to implementation, and changes with implementation over time. Interviews were audio recorded, transcribed verbatim, and anonymized.

As part of the larger study, we conducted observations of safety meetings and activities in the units and among hospital safety leadership to attain an in-depth understanding of safety practices. These observations consisted of events where CLABSI appeared as a topic of conversation (e.g., continuous improvement huddles, safety coach meetings, executive quality meetings) as well as events where CLABSI was the specific focus (e.g., CLABSI rounds and audits). The observations were ethnographically informed whereby we were attentive to details such as space, people, objectives, interactions, activities, time, goals, and feelings [38]. Prior to each observation, participants were informed about the study and purpose of observations, and given the opportunity to ask questions or express to not be observed. The researchers recorded notes during the observations and transcribed them following the session, adding descriptive details and analytical interpretations. No participant identifying information was recorded.

We also collected documents such as hospital strategy documents, safety coach reports, and hospital communication about safety stories and outcomes.

We used a reflexive thematic analysis [39] approach that involved stages of generating initial codes, searching for themes, reviewing themes, and defining and naming themes. We coded the data manually (using Microsoft Word). The coding framework and themes were initially informed by a conventional content analysis approach, being derived directly from the data. We then used a directed approach, where we used social science literature on bundle and guideline implementation to sensitize us to social processes influencing CLABSI bundle implementation (e.g., individuals’ interpretations and responses to risk and safety; professional socialization and boundaries; social practices of safety data reporting; interactions between individual actions and organizational and structural factors). Analysis and interpretation of interview, observation, and documentary data was guided by the constant comparison method [40] as we moved back and forth within and between the data collected from the hospital and safety leadership and three units. Method and data triangulation allowed the phenomenon of central-bundle implementation to be examined through different perspectives. Researcher triangulation occurred given that the research team discussed the codes and themes as they developed, and implications of the findings, to enable rigor in the analysis and interpretation. During analysis, we were reflexive of our disciplinary and professional backgrounds and roles related to leading and studying the hospital safety program, clinical work, and quality and safety research.

## Results

We conducted 74 interviews (mean 45 min, range 26–74 min) with 71 participants with different professional backgrounds and roles. At the organizational level, participants included hospital executives and safety leadership. At the unit level, participants included those with varied professional backgrounds (e.g., nursing, medicine, pharmacy) and who worked in clinical, managerial, education, and QI roles. Interviews were one-on-one except for two with two or three people. Six hospital executive and safety leaders were interviewed a second time nearer the end of data collection as their interviews occurred in the initial stage of the hospital safety program and we thought it could be useful to also interview them at later stages of implementation. We undertook approximately 50 h of observations. The findings are organized in three sections, based on the themes developed through data analysis, that demonstrate different ways that socio-cultural factors influenced CLABSI bundle implementation: variable belief in CLABSI as an improvement target, variation in unit-specific bundle practices and priorities, and complexities of assessing and monitoring adherence.

### Variable belief in CLABSI as an improvement target

The hospital identified CLABSI prevention as an organizational priority with the expectation that all units would similarly prioritize it as a QI target. Hospital leadership conveyed this priority in hospital communications. For example, the 2017–2018 safety progress report stated:

*Our focus on reducing hospital acquired conditions is relentless. We’re particularly focused on reducing the number of CLABSIs and SSIs (surgical site infections) patients experience … as these … represent a high proportion of our preventable conditions.* (p. 10)

Study participants did not appear to fully share this prioritization, as they expressed different preferences based on their professional roles and experiences. Some participants expressed reservations about investing resources in CLABSI prevention because the CLABSI rate on their unit was low or there was little data supporting a problem. This perception was expressed by a physician in the medical specialty unit:

*So, I’m not a big believer … I think we have overshot the bundle stuff because once those lines are healed in, really it’s not that common that they got infected. But everyone feels very good about it so I don’t argue…* (Physician, medical specialty)

The surgeons interviewed noted that if there was a problem with CLABSI, they were already doing what they needed to do, and, in most cases, other healthcare professionals were more directly responsible for its prevention. In particular, surgeons explained that they already avoid central lines if possible. Furthermore, if lines are put in, insertion is done by interventional radiology, nurses handle the lines, and other physicians monitor patients with them:

*We’re always thinking when can we take out that central line? I haven’t seen any big data on the ward about our CLABSI rates … I know there is a bundle … and the nurses are supposed to follow it. That I’m aware of but if you ask me what are our CLABSI rates I couldn’t tell you.* (Physician, surgery)

Others, particularly in the medical specialty unit, did agree that their CLABSI rate was high. However, some viewed that rate as a natural function of their patient population that would make it unresponsive to a QI intervention, and were also concerned about the reliability of CLABSI report data:

*There are some oncology providers who believe that CLABSI is something that we should expect in oncology. They all have central lines and many of them don’t have any neutrophils, so they will get a CLABSI. So, that has been a big education point … People are coming around. Convincing people that zero is achievable is very difficult, but there’s buy-in now that we can do better. That’s a good start …* (Safety leader, medical specialty)

In contrast, ICU participants welcomed the hospital’s focus on CLABSI prevention as it supported their prioritization of this goal several years earlier. ICU staff, though, had divergent perspectives from hospital leadership about *how* to achieve CLABSI reduction goals, some viewing the new bundle as being “less rigorous than we had been” (Physician, ICU). ICU staff reported that their patient population is at higher risk and therefore required a stricter protocol for central line care.

*We had concerns about changing practice so that the practice was the same across the hospital. Totally understand about why we need to have the same practices … The problem is, is that neonates are different and especially preterm babies and I know there are neonates in the rest of the hospital. But I felt that maybe our voice was not being completely heard in terms of our concerns and so, we did our best to identify that as well as listen to their recommendations and we made changes.* (Nurse, ICU)

Nursing leaders in the units studied were not as focused on whether or not CLABSI should be an improvement target, but were more concerned about CLABSI being one of an overwhelming number of QI targets for which they were responsible. They struggled with not having sufficient resources to respond to numerous top-down QI initiatives.

*I don’t know that there always is a true understanding of what it’s actually like at the front line. Just in terms of the things that they’re asking nurses to do, it feels like we’re always adding extra things, and we’re not taking anything away. And so, they think, oh well, it’s just one more thing. It’s just this small little thing … it’s only five checks and it only takes five seconds. But, when we’ve added 100 things that are five seconds, it takes a lot of time.* (Educator, surgery)

Given the variable support among staff for CLABSI as an improvement target, hospital safety leaders had to work over time with each unit according to their responses to the organizational CLABSI prioritization. However, as the leaders noted, there was also tremendous variability within each unit:

*Some teams dive right in. When you talk about the focus needing to be bundle adherence, the why is clear, they understand what their part is and that’s … what we need to understand more … is what makes a unit culture thrive and dive right into CLABSI … .versus other units where a lot of my work is proving that it’s important work that we need to do or proving or answering questions related to the why, mostly.* (Hospital safety lead)

### Variation in unit-specific bundle practices and priorities

The messaging around CLABSI prevention focused on the implementation of standard practices across the hospital, as expressed below by a safety leader during a meeting:

*Each [hospital-acquired] condition has its own set of practices and the belief is that if applied the same way for every patient, then we will be able to get to zero.* (Observation, 092718)

However, findings revealed challenges to achieving bundle practice standardization.

Education was the primary strategy to support bundle implementation, with each unit responsible for doing this education initially. This put significant pressure on units as they had to figure out how to deliver education for nurses given competing requirements (other education, patient care demands). They adapted by using their limited education days or one-on-one teaching by unit nursing leadership. Hospital safety leadership recognized that there were inconsistencies in what was being taught and how terms such as “aseptic non-touch technique” were being interpreted. They therefore developed centralized education such as a website with learning material and resources, and organized reinforcement tactics such as a common cart with materials for standardized dressing changes and had clocks installed to support timing requirements. Safety leaders reported progress over time to standardize practices such as nurses’ increased use of print or online bundle resources, and having discussions to anticipate the procedure and manage the physical environment ahead of time.

The findings demonstrated, though, that behavioral changes were contingent on factors beyond knowledge and equipment availability. ICU nurses, for example, described challenges enacting the recommended two-person approach to line care procedures, given patient care needs and family visiting patterns:

*If you have six babies in one room and there’s three nurses, and all of them have central lines. And all of your TPN (total parenteral nutrition) and lipids come up at 5:00 and your handover’s at 7:00, but all of your babies need to be done up and fed at 6:00. And you’ve got meds, and parents are usually visiting, then it’s a really busy time and there isn’t another person to help you … when you start adding additional components, sometimes it’s tough to follow through with exactly what you need to do…* (Nurse, ICU)

Nurses also had to make decisions about competing patient care priorities. For example, medical specialty nurses described the tensions between CLABSI bundle requirements and patient and family preferences:

*If the family gets it, and they are terrified of their kid getting an infection, then they’ll do what we ask. If they prioritise it differently in the sense that they’re more focused on the fact that their child doesn’t feel very well today, and they don’t like baths anyway, so I’m not going to do the bath because that’s just going to make them feel even worse, it’s difficult … Our staff are pretty good at offering … Trying to make it as easy as possible. But sometimes for some families, the most that they can accomplish is just physically being here and not falling apart because they’re just so stressed.* (Safety lead, medical specialty)

The nurses received education about the CLABSI bundle requirement for a standard daily discussion among nurses and other interprofessional team members around three questions: (1) line necessity, (2) functionality, and (3) utilization. However, nursing abilities’ to initiate and discuss these questions required more than changes in an individual nurse’s knowledge and practice, as it was contingent on each unit’s existing rounding routines and interprofessional interaction patterns. In the ICU, physician safety leadership, an established practice of questioning around the line, and creation of a rounding sheet to structure interactions, facilitated nursing input into standard daily discussions. The medical specialty unit also had interprofessional rounds; however, participants explained that the three required questions concerning line necessity, functionality, and utilization did not fit their patient care practices. The unit therefore adapted the line discussion to focus more on how often the line is accessed as opposed to whether it continues to be required. This change, though, continued to require ongoing attention:

*But then how do we talk about a line in a way that’s meaningful for staff? How often are we doing blood work? Can we switch anything to oral? Is [the line] working well?… so those [types of] conversations. We have to shift a little bit of that, not necessarily away from the bundle, but shift it in a way that it made meaning for staff … That is still where we struggle the most, is that discussion, and having it happen on a consistent basis. We’re really good about talking about lines when they’re not working well, not so much when they’re working well …* (Safety lead, medical specialty)

Nurses in surgery also expressed some resistance to the bundle questions. For example, they viewed the line discussion to be redundant:

*… it’s tough, because of your clinical judgement, you know if this patient has a stricture they’re going to the OR in a week, they’re staying NPO for a week before they go to the OR. Of course it’s a necessity. We don’t need to be asking that question every day when we know there’s a plan.* (Safety lead, surgery)

However, there was not the same investment of attention as in the medical specialty unit to strategizing how the surgical unit could adapt the line discussion. This was compounded by the surgical unit not having routine interprofessional rounding routines, but rather variable rounding practices of individual surgeons. Nurses and physicians both described the variability in opportunities for interprofessional interactions in this context.

Over time, as the variability of units’ bundle practices became apparent, hospital leadership worked to address the emerging issues by meeting with the range of relevant stakeholders “to have different groups understand what their role is” (Hospital safety lead). However, they also noted the resources and time required. This led some hospital safety leaders to recognize limitations to a top-down approach that expects all units to perform the bundle in a uniform way:

*… in the early days of spreading the HAC bundle, there was a desire to spread the bundle as it is, but then an acceptance of unit and local contextual interpretation … there has been a lot of conversation about this balance between having a standard approach, which means everybody does the same thing at all times, regardless of where the location is, versus those adaptive changes to fit an environment.* (Hospital executive)

### Complexities of assessing and monitoring adherence

Audits assessed bundle adherence and by extension were expected to correspond to CLABSI rates. Auditing was not a straightforward assessment process because each unit conducted their own audits and these were subject to personnel and resources available in the unit. In addition, responsiveness to being audited and audit results differed based on who was doing the auditing such as a nurse champion, manager, or QI leader.

Unit QI leaders tended to describe the audit as an opportunity to provide teaching and guidance. In the ICU, for example, clinical navigators support new staff, and therefore being audited by them was viewed as beneficial by providing a second person to help with the cap or line change and correct technique and therefore “you almost wanted to be audited” (Nurse, ICU). In contrast, peer to peer auditing in the ICU was described by a participant as a routine, check box activity. These different approaches are apparent in the quotes below:

*… I’m not fixated on we need to do 20 (audits) and check off boxes. I’m fixated on it being a meaningful and deep conversation around what could have done better so that I’m hardwiring practice.* (Unit leadership, medical specialty)

*I think audits are a good idea in theory, but, full disclosure, if I’m auditing my roommate, we’re friends, we’re colleagues, am I watching literally every minutia she’s doing? Probably not. I’m giving her the checks. Right? So, does that necessarily negate the efficacy of the audits? Probably. A little bit … everything’s supposed to be a two-person procedure for CLABSI. Do we always have time for that? No. So, we do our best, but it’s also kind of that marrying of reality and expectations.* (Nurse, ICU)

The auditing tool was also used differently depending on the individual, as auditors distinguished between “green” versus “red” when an action was not initially performed but then consequently completed, resulting in variability in the assessments. Relatedly, audit results were not seen by hospital safety leadership as correlated with CLABSI rates, and therefore not helping to identify where the gaps were. In response to reliability concerns, unit-based auditing was supplemented by three central hospital auditors, who received the same training to develop a consistent approach to completing the audit form and providing feedback to nurses. However, even these auditors encountered dilemmas given the ambiguity and complexity of bundle practices and their documentation. In some instances, nurses documented earlier in a day in anticipation of a bath being done or a team discussion happening, but one could not be certain whether they actually occurred. There was also the possibility that nurses did not document a required action but had done it (Observation, January 22, 2019).

The centralized auditing approach demonstrated lower levels of bundle adherence and was therefore perceived to improve measurement reliability; however, other factors affected central auditors’ abilities to audit, specifically their varying interactions with each of the units. While the auditors had organizational support, units exerted control over access. Some units collaborated with the central auditors to supplement their own efforts, while others were more resistant to them coming into their units.

*And I think honestly the main difference is the cultures on those units are just so different … So, how they receive audit, how they are open or not to our team coming in and doing practice observations has looked very, very different …* (Hospital safety lead)

The nurses had varied perspectives about being audited, which influenced their responsiveness to, and engagement with, the auditing process, and also morale. For some, auditing was perceived as useful feedback to help them with their practice. Others viewed the auditing as problematically targeting nurses when bundle practice is dependent on a wider group of healthcare providers.

## Discussion

This study provides insight into the trajectory of a hospital-wide implementation of a central-line bundle among three units within a hospital which ultimately achieved its measurement outcome goals. The themes of variability in beliefs about CLABSI as an improvement target, in unit-specific bundle practices and priorities, and in assessment and monitoring of bundle adherence, contribute to our understanding of how clinically contextual factors influence CLABSI prevention practices. These findings highlight the opportunities and challenges of balancing central versus unit-based approaches, and standardization versus adaptation, of bundle practices in organization-wide improvement [41–43].

Our findings extend understandings of socio-cultural factors influencing central-line bundle implementation, addressing gaps in understanding how clinical contexts influence the nature and effectiveness of improvement interventions [44, 45]. The significance and variability of socio-cultural contextual factors were observed across beliefs of CLABSI as an improvement target, performance of bundle requirements, and auditing practices. In some cases, these findings are explicitly linked to the clinical nature of the unit, such as the care needs of the patient population of the unit (e.g., oncology patients’ susceptibility to CLABSI) and workflow patterns (e.g., surgeons not being in the unit during the day). Other contextual factors, such as resources for auditing and existing educational structures, are less connected to unit clinical characteristics, but distinctive unit particularities shape the attention given to bundle practices. Professional issues such as roles and interactions are influenced by a range of factors, including clinical contexts [46, 47]. Important factors for bundle implementation in our study, which played out differently across units, included nurses and physicians’ interpretations of their roles in relation to CLABSI prevention and existing interprofessional communication and rounding practices that enabled or challenged opportunities for central-line discussions.

At our study site, safety leaders made efforts to respond to socio-cultural factors influencing bundle implementation, which involved shifting between centralized and unit-based approaches. Our findings confirmed the importance of hospital prioritization of CLABSI prevention, a noted implementation facilitator [48]. This organizational-level priority gave CLABSI prevention work legitimacy at the clinical level, and allowed individuals within the unit to leverage this organizational mandate to support unit efforts. However, when CLABSI was not initially recognized as a problem worth targeting, the organizational priority setting was less impactful, and changes took longer to achieve impact; underlining the need for senior leadership to do more than identify a safety problem, they need to have bottom-up engagement in shared construction of the problem and solutions [49]. This insight was only recognized and acted upon over time. When pursuing a bottom-up approach (i.e., unit-based education and auditing), local resource constraints and unit-specific approaches led to variation in implementation. These challenges (and the perception that auditing did not help to reduce CLABSIs) prompted adopting a centralized approach to education and auditing along with regular leader CLABSI-focused rounding. Still, some socio-cultural factors such as professional interactions were more challenging to address and were not visibly done so at either level. Tensions between local and centralized approaches, as in the case of central auditors accessing units, continued to exist. CLABSI prevention targets were achieved despite less attention to these issues. However, this achievement does not detract from their likely importance to sustainable and widespread improvement [7], and the importance of attending to what is, and is not, captured through measurements in QI programs. Our findings reflect the need for continual attention to balancing and maintaining top level commitment in CLABSI prevention and local ownership in QI initiatives, as well as to complex patterns of professional interactions, unit norms, and hierarchical relationships [17, 50, 51].

A related, but different, issue in our findings was the tension on standardization versus customization of bundle practices. The emphasis on “standardization” in QI is challenged when standardized practices are being placed into spaces with pre-existing norms, practices, and procedures [52]. In our study, despite an organizational emphasis on standardization, in practice, each unit was making adaptations relevant to their contexts, with recognition of the possible need for adaptation becoming apparent to hospital leadership over time. Unit leadership played key roles in bundle adoption and adaptation, as was apparent in the efforts by ICU leadership to engage in discussions and negotiations with hospital leadership about bundle practices, and by medical specialty unit leadership who supported the adaptation of central-line related questions during the rounds. These findings connect back to initial bundle guidelines allowing for local customization and appropriate clinical judgment [1], and the Michigan Keystone project reporting there being not one “Keystone checklist,” but more than 100 versions [13]. Whereas in our study, recognition of the possibility for customization only emerged over time and was not systematic, creating space and resources to allow for discussions about “non-negotiable elements” vs. what can be locally customized [53] might require more purposeful attention in improvement programs.

Our study has limitations. The data reflect three units in one Canadian hospital which may limit the transferability of findings, as clinical and hospital contexts differ. However, the nature of our findings are consistent with results from other implementation studies, and, additionally, the findings are important from a conceptual generalizability perspective, in terms of how they can inform other healthcare settings [54]. Our methodological approach, and the separateness of the qualitative study from the quantitative hospital measurement process, did not allow for a closer examination of our findings and unit-specific CLABSI measurements over time. Data collection occurred from April 2017 through February 2019 and changes in implementation processes are likely to have continued to occur after this period of time.

## Conclusions

Our qualitative findings, in addition to hospital CLABSI measurement outcomes, demonstrate the efforts required to implement a central-bundle. They also reinforce the importance of moving from a narrow focus on intervention fidelity to viewing bundle implementation as an emergent, dynamic, and long-term process that attends to the reciprocal interaction between an intervention and its unit and hospital socio-cultural contexts [51, 55, 56]. This approach allows for a focus on CLABSI to be positioned over time in relation to other unit and hospital quality priorities [57]. Implementation approaches need to balance top-down support and bottom-up adaptation. However, we also note that despite earnest implementation efforts and successful measured outcomes, our findings show that certain challenges such as competing quality priorities, limitations of interprofessional interactions, and systematic auditing were not comprehensively addressed. We recognize that attention to these socio-cultural factors is likely essential to broader strengthening of organizational capability [7, 53]. CLABSI reductions should be celebrated; however, we need to continue to be mindful of safety issues requiring attention in addition to these measured outcomes. We hope our work helps sharpen the discussion and decision-making about how to allocate QI resources and ensuring the consideration of socio-cultural factors in achieving quality goals.

## Data Availability

The datasets generated and/or analyzed during the current study are not publicly available due to the confidentiality of qualitative and hospital safety data collected.

## Abbreviations

CLABSI: Central-line-associated bloodstream infections
QI: Quality improvement
IHI: Institute for Healthcare Improvement
ICU: Intensive care unit
SPS: Children’s Hospitals’ Solutions for Patient Safety Network
HAC: Hospital-acquired condition
MBI: Mucosal barrier infection
NHSN: National Healthcare Safety Network
